# Non-pneumatic anti-shock garment for improving maternal survival following severe postpartum haemorrhage: a systematic review

**DOI:** 10.1186/s12978-015-0012-0

**Published:** 2015-03-31

**Authors:** Cynthia Pileggi-Castro, Vicky Nogueira-Pileggi, Özge Tunçalp, Olufemi Taiwo Oladapo, Joshua Peter Vogel, João Paulo Souza

**Affiliations:** Department of Pediatrics & Glide Technical Cooperation and Research, Ribeirão Preto Medical School, University of São Paulo, São Paulo, Brazil; UNDP-UNFPA-UNICEF-WHO World Bank Special Programme of Research, Development and Research Training in Human Reproduction, Department of Reproductive Health and Research, WHO, Geneva, Switzerland; Department of Social Medicine, Ribeirão Preto Medical School, University of São Paulo, Av. Bandeirantes, 3900 Ribeirão Preto, SP Brazil

**Keywords:** Postpartum haemorrhage control, Non-pneumatic anti-shock garment, NASG, Maternal mortality reduction

## Abstract

**Introduction:**

Women with postpartum haemorrhage (PPH) in developing countries often present in critical condition when treatment might be insufficient to save lives. Few studies have shown that application of non-pneumatic anti-shock garment (NASG) could improve maternal survival.

**Methods:**

A systematic review of the literature explored the effect of NASG use compared with standard care for treating PPH. Medline, EMBASE and PubMed were searched. Methodological quality was assessed following the criteria suggested by the Cochrane Effective Practice and Organization of Care Group. Guidelines on Meta-analysis of Observational Studies in Epidemiology were used for reporting the results. Mantel-Haenszel methods for meta-analysis of risk ratios were used.

**Results:**

Six out 31 studies met the inclusion criteria; only one cluster randomized controlled trial (c-RCT). Among observational studies, NASG fared better than standard care regarding maternal mortality reduction (Relative Risk (RR) 0.52 (95% Confidence interval (CI) 0.36 to 0.77)). A non-significant reduction of maternal mortality risk was observed in the c-RCT (RR: 0.43 (95% CI: 0.14 to 1.33)). No difference was observed between NASG use and standard care on use of blood products. Severe maternal outcomes were used as proxy for maternal death with similar pattern corroborating the trend towards beneficial effects associated with NASG.

**Conclusion:**

NASG is a temporizing alternative measure in PPH management that shows a trend to reduce PPH-related deaths and severe morbidities. In settings where delays in PPH management are common, particularly where constraints to offer blood products and definitive treatment exist, use of NASG is an intervention that should be considered as a policy option while the standard conditions for care are being optimized.

## Introduction

Maternal mortality is a public health indicator related to social development and health equity around the world and one of the main indicators for monitoring the progress toward the Millennium Development Goal 5 (MDG-5). According to global estimates, haemorrhage accounted for 661,000 maternal deaths around the world between 2003 and 2009 which represents about 27% of all maternal deaths [1]. Most of these deaths are avoidable and take place in low-income countries [2]. Severe postpartum haemorrhage (PPH) is a leading cause of these deaths and is defined as a condition of maternal active genital bleeding after delivery, with at least one of the following: perceived abnormal bleeding (1000 mL of more) or any bleeding with hypotension or blood transfusion [3]. The use of uterotonics following cord clamping can reduce the occurrence of postpartum haemorrhage [2,3]. In addition to prophylactic uterotonics, growing evidence suggest that appropriate management of PPH could prevent maternal deaths related to severe PPH. Appropriate PPH management includes a rapid recognition of persistent blood loss after initial interventions such as uterotonics and fluid resuscitation [2,4]. Continuous monitoring and assessment of the woman just after delivery would allow implementation of appropriate interventions to reduce blood loss should PPH occur. However, in many settings around the world, delays during PPH prevention, diagnosis and treatment occur and contribute to avoidable maternal death and morbidity [5].

The Non-pneumatic Anti-Shock Garment (NASG) is a device developed as a temporizing measure to regain hemodynamic stability and allow patient transfer or definitive PPH treatment. The NASG is a compression suit made of five neoprene segments that close tightly with Velcro around the legs, pelvis and abdomen [6]. The lower body circumferential pressure made by the device shunts blood from the lower extremities and abdominal area to the essential core organs: heart, lungs, and brain. Within minutes of application, women suffering from shock have been seen to regain consciousness, normalize their vital signs and reduce the blood loss [7,8]. An observational study suggested that NASG use adds some time for postpartum haemorrhage women until definitive treatment can be reached, especially in other aetiologies rather than ruptured uterus [9]. In five quasi-experimental, non-randomized studies, NASG intervention at tertiary facilities was associated with reduced odds of death for women with hypovolemic shock secondary to obstetric hemorrhage [10]. Lastly a qualitative study in rural areas in Mexico suggested positive responses on acceptability of health personel to NASG [11]. Fostered by the observational trends of effect on NASG use for improving maternal health conditions. We conducted a systematic review to assess the effectiveness and safety of using NASG in improving maternal outcomes following severe PPH in settings where recourses are scare or definitive treatment to PPH may be delayed.

## Methods

We conducted a systematic review of the literature following the Meta-Analysis Of Observational Studies in Epidemiology guidelines and the Preferred Reporting Items for Systematic reviews and Meta-Analyses statement with a pre-specified protocol for data collection [12,13]. The standard care for treating PPH is defined by WHO guideline consisting on using uterotonics, uterine massage and fluid resuscitation with crystalloids [2]. All studies presenting comparison between standard care for treating hypovolemic shock secondary to PPH and standard care plus NASG application were eligible to this systematic review. Data collection process followed a standard collection form. The outcomes of interest were maternal mortality, severe maternal outcomes defined by the severe maternal morbidity plus maternal deaths, maternal side effects requiring treatment and the use of blood products.

An electronic search was conducted in March 2014 using the following online databases: Medline, EMBASE and PubMed. The search strategy used was NASG [All Fields] OR (non-pneumatic [All Fields] AND anti-shock [All Fields] AND (“clothing”[MeSH Terms] OR “clothing” [All Fields] OR “garment”[All Fields])). All studies identified through the electronic search had their titles and abstracts examined. Publications with incomplete reporting results and non-intention to treat analysis were excluded. Full texts of eligible studies were retrieved and assessed without language restrictions. The reference list of eligible studies was also screened to identify other potentially relevant paper to this review. Leading researchers in the field were contacted in the search for additional studies, particularly unpublished ones. Two reviewers assessed all eligible studies and a third reviewer was consulted in the cases of disagreement (CPC). Quality assessment of the studies was conducted based on Cochrane Effective Practice and Organisation of Care Group guidelines [14], Strengthening the Reporting of Observational studies in Epidemiology [13] and Consolidated Standards of Reporting Trials (CONSORT: extension to cluster randomized trials) [15]. Single data extraction was conducted (VNP) and checked by a second reviewer (JPS). Authors of included studies were contacted for providing data on outcomes of interest not included in the published reports.

Review Manager 5 Software (RevMan, computer program. Version 5.2. Copenhagen: The Nordic Cochrane Centre, The Cochrane Collaboration, 2012) was used for quantitative analysis [16]. Random effect Mantel-Haenszel methods for meta-analysis of risk ratios were used to produce pooled estimates of effect on NASG use for controlling PPH. Heterogeneity was measured with I^2^ test. Grading of the quality of evidence and the strength of recommendations tables were produced in order to inform decision making process for developing recommendations once it combines transparency in judgments of the best available evidence, with regards on the measure of effect, quality and assessment of important outcomes for end users of the intervention (GRADE software, Version 3.6, 2011 update) [17]. Outcomes selected by the 2012 WHO Postpartum haemorrhage guidelines were used for assessing the quality of evidence generated by included studies [2].

## Results

**Figure 1:**
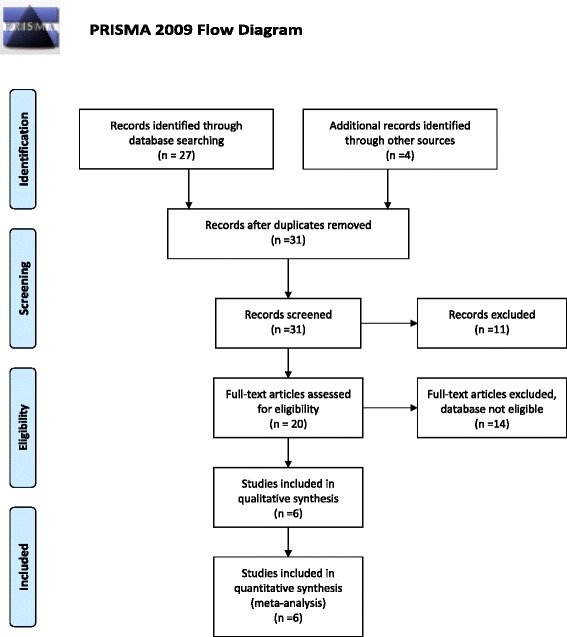
**Flow diagram of the systematic review.**

The electronic search strategy produced a total of 27 citations and additional four records were identified through other sources, such as personal communication with experts and reference lists review. Among the 31 records that had the titles and abstracts screened, 11 were excluded because were considered as clearly not relevant. Twenty full text articles were retrieved and assessed for eligibility. Fourteen studies were excluded because they reported on different analysis of the same data or because there was no comparison with standard treatment. Only six studies met the inclusion criteria of this systematic review, five observational studies (four before-after, one non-randomized clinical trial) and one cluster randomized trial (cRCT) [18-23]. There was no report on side effects of the intervention requiring treatment. Figure 1 shows the PRISMA flow diagram for the selection of eligible studies [12]. Table 1 presents the characteristics of included studies with risk of bias assessment of each study and Table 2 shows excluded studies (link to Tables 1 and 2). Pooled analysis proceeded for observational studies.

**Table 1:** **Characteristics of included studies**

**Miller et al., 2006** [18]	
Study design	Controlled before-after
Population	Women with obstetric haemorrhage and signs of shock (>750 mL of blood loss and either pulse of >100 beats per minute or systolic blood pressure of <100 mmHg)
Intervention	Standard care versus standard care plus NASG.
	Standard protocol: “administration of crystalloid intravenous fluids, use of uterotonic medications (IV oxytocin and rectal misoprostol), uterine massage, determining the source of bleeding, and providing blood transfusions and surgery as necessary. Vaginal procedures included manual removal of the placenta, suturing of lacerations, manual vacuum aspiration or suction curettage, and uterine evacuation using ring forceps (also known as ‘sponge sticks’ or ‘sponge holders’). Surgery was performed when the haemorrhage continued despite the other interventions. Practitioners used a variety of surgical interventions (step-wise ligation of uterine arteries, B-Lynch suture, or hysterectomy) according to their experience and the clinical situation. The only difference in care for the women in the NASG group was that the NASG was placed on the woman when she met study entry criteria. The NASG was left in vaginal procedures.”
Outcomes	Maternal mortality, maternal morbidity, overall blood loss
Setting	Four university teaching facilities in Egypt
Quality assessment	GRADE quality of evidence: Low, downgraded for study design, without serious limitations in risk of bias, inconsistency, and indirectness or imprecision.
	EPOC methods assessment: low risk of bias considering study design.
**Miller et al., 2010** [19]	
Study design	Controlled before-after
Population	All “pregnant (with a non-viable fetus), birthing, or postpartum women experiencing hypovolemic shock secondary to obstetric haemorrhage from any aetiology. Additional inclusion criteria were estimated blood loss of at least 1000 mL and/or at least 1 clinical sign of hypovolemic shock (systolic blood pressure 100 mm Hg or pulse 100 beats per minute)”.
Intervention	Standard care plus NASG versus standard care.
	Standard care consisted on “administration of crystalloid intravenous fluids (≥1500 mL in the first hour following study admission); administration of uterine massage and uterotonic medications for uterine atony (intravenous or intramuscular oxytocin, intramuscular ergometrine, and rectal misoprostol); vaginal procedures; provision of blood transfusions (standard in the 2 study sites for women who lost ≥1000 mL of blood); and surgery. The NASG was left in vaginal procedures”.
Outcomes	Extreme adverse outcomes combined with maternal mortality and morbidity; secondary outcomes (overall blood loss, urine output, emergency hysterectomy for uterine atony)
Setting	Two tertiary hospitals Egypt
Quality assessment	GRADE quality of evidence: Low (downgraded by study design, without serious limitations in risk of bias, inconsistency, indirectness or imprecision).
	EPOC methods assessment: low risk of bias considering study design.
**Ojengbede et al., 2011** [20]	
Study design	Controlled before-after
Population	Woman with post-partum haemorrhage (initial blood loss of ≥750 ml) due to uterine atony, retained placenta, ruptured uterus, vaginal and cervical lacerations or placenta accreta and one clinical sign of shock (systolic blood pressure <100 mmHg or pulse >100 beats/min).
Intervention	Standard care plus NASG versus standard care.
	Standard care: “administration of crystalloid intravenous fluids (≥1,500 ml in the first hour), uterotonic medications (oxytocin, ergometrine, syntometrine, misoprostol), uterine massage for patients with uterine atony, vaginal procedures (bimanual compression, manual removal of placenta or dilation, repair of lacerations and curettage for retained tissue) and abdominal surgeries (arterial ligation, B-Lynch compression sutures, hysterectomy) as necessary”.
Outcomes	Maternal mortality and overall blood loss
Setting:	Four tertiary facilities in Nigeria (2 teaching and 2 state facilities)
Quality assessment	GRADE quality of evidence: Low (downgraded by study design, without serious limitations in risk of bias, inconsistency, indirectness or imprecision).
	EPOC methods assessment: low risk of bias considering study design.
**Maknikar et al., 2012** [22]	
Study design	Non randomized clinical trial
Population	Woman with post-partum haemorrhage and signs of hypovolemic shock
Intervention	Standard care plus NASG versus Standard care
Outcomes	Maternal mortality, maternal morbidity, overall blood loss
Setting	Fifteen facilities India
Quality assessment	GRADE quality of evidence: Low (downgraded by study design, without serious limitations in risk of bias, inconsistency, indirectness or imprecision).
	EPOC methods assessment: high risk of bias as stated “lack of randomization”
	Only abstract available with complementary information provided by contact author
**Magwali et al., 2012** [21]	
Study design	Before-after
Population	Woman with post-partum haemorrhage and signs of hypovolemic shock
Intervention	Standard care versus standard care plus NASG
Outcomes	Maternal mortality and overall blood loss
Setting	Two hospitals Zimbabwe
Quality assessment	GRADE quality of evidence: Low (downgraded by study design, without serious limitations in risk of bias, inconsistency, indirectness or imprecision).
	EPOC methods assessment: low risk of bias considering study design.
	Only abstract available with complementary information provided by contact author.
**Miller et al., 2013** [23]	
Study design	Cluster randomized controlled trial
Population	Woman with obstetric haemorrhage from any aetiology and hypovolemic shock before removal from primary health care centres to higher level complexity of care facility, with at least two of the following eligibility criteria: visually estimated blood loss >500 mL, pulse > 100 BPM, systolic blood pressure <100 mm Hg.
Intervention	Standard care plus NASG application versus standard care
	“Standard shock/haemorrhage protocol: oxygen, IV fluids, uterotonics/uterine massage (for uterine atony), suturing of lacerations, manual removal of placenta or retained tissues, MVA, surgery, and blood transfusion, as necessary. The only differences in treatment received depended on haemorrhage aetiologies”.
Outcomes	Maternal mortality rates; survival with severe maternal morbidity; and extreme adverse outcome. As secondary outcomes included median blood loss measured by weighing the absorbent pad(s) upon admission; blood loss measured in the drape at arrival; blood loss during surgery; frequency of emergency hysterectomy for intractable uterine atony; and time to recovery from shock (defined as return to Shock Index (SI) 0.98 (SI = Heart Rate/Systolic Blood Pressure) as well as negative effects that might be attributable to the NASG application (decreased urine output, respiratory difficulties, nausea, vomiting and abdominal pain).
Setting	Primary health care services (38) in Zambia and Zimbabwe
Quality assessment	GRADE quality of evidence: Low (downgraded because imprecision, few events). No other limitation found.
	EPOC methods assessment: low risk of bias considering study design.

**Table 2:** **Excluded studies listed by reasons**

**Excluded papers for not being related to non-pneumatic garment tests**	
1	Di YP. Functional roles of SPLUNC1 in the innate immune response against Gram-negative bacteria. Biochemical Society transactions. 2011;39 (4):1051–5.
2	Gates AJ, Luque-Almagro VM, Goddard AD, Ferguson SJ, Roldan MD, Richardson DJ. A composite biochemical system for bacterial nitrate and nitrite assimilation as exemplified by Paracoccus denitrificans. The Biochemical journal. 2011;435 (3):743–53.
3	Hauswald M, Williamson MR, Baty GM, Kerr NL, Edgar-Mied VL. Use of an improvised pneumatic anti-shock garment and a non-pneumatic anti-shock garment to control pelvic blood flow. International journal of emergency medicine. 2010;3 (3):173–5.
5	Podymova SD. [The evolution of ideas about nonalcohol fatty liver disease]. Eksperimental’naia i klinicheskaia gastroenterologiia = Experimental & clinical gastroenterology. 2009 (4):4–12.
6	Lateef F, Kelvin T. Military anti-shock garment: Historical relic or a device with unrealized potential? Journal of emergencies, trauma, and shock. 2008;1 (2):63–9.
7	Miller S, Martin HB, Morris JL. Anti-shock garment in postpartum haemorrhage. Best practice & research Clinical obstetrics & gynaecology. 2008;22 (6):1057–74.
8	Geller SE, Adams MG, Miller S. A continuum of care model for postpartum hemorrhage. International journal of fertility and women's medicine. 2007;52 (2–3):97–105.
9	Liu ZQ, Tian YQ, Peng C, Hu YF, Zhou M, Ouyang J, et al. Expression of NASG gene and its role in human nasopharyngeal homogenous tissue cells. Chinese medical journal. 2005;118 (13):1076–80.
10	Zhang B, Nie X, Xiao B, Xiang J, Shen S, Gong J, et al. Identification of tissue-specific genes in nasopharyngeal epithelial tissue and differentially expressed genes in nasopharyngeal carcinoma by suppression subtractive hybridization and cDNA microarray. Genes, chromosomes & cancer. 2003;38 (1):80–90.
11	Zhang BC, Zhu SG, Xiang JJ, Zhou M, Nie XM, Xiao BY, et al. [Analysis of splicing variants in NASG 3′UTR, down-regulated in nasopharyngeal carcinoma, and its expression in multiple cancer tissues]. Ai zheng = Aizheng = Chinese journal of cancer. 2003;22 (5):477–80.
**Excluded papers because they were different analysis of the same databases of included studies**	
1	Sutherland T, Downing J, Miller S, Bishai DM, Butrick E, Fathalla MM, et al. Use of the non-pneumatic anti-shock garment (NASG) for life-threatening obstetric hemorrhage: a cost-effectiveness analysis in Egypt and Nigeria. PloS one. 2013;8 (4):e62282.
2	Fathalla MM, Youssif MM, Meyer C, Camlin C, Turan J, Morris J, et al. Nonatonic obstetric haemorrhage: effectiveness of the nonpneumatic antishock garment in egypt. ISRN obstetrics and gynecology. 2011;2011:179349.
3	Turan J, Ojengbede O, Fathalla M, Mourad-Youssif M, Morhason-Bello IO, Nsima D, et al. Positive effects of the non-pneumatic anti-shock garment on delays in accessing care for postpartum and postabortion hemorrhage in Egypt and Nigeria. Journal of women’s health. 2011;20 (1):91–8.
4	Miller S, Fathalla MM, Ojengbede OA, Camlin C, Mourad-Youssif M, Morhason-Bello IO, et al. Obstetric hemorrhage and shock management: using the low technology Non-pneumatic Anti-Shock Garment in Nigerian and Egyptian tertiary care facilities. BMC pregnancy and childbirth. 2010;10:64.
5	Mourad-Youssif M, Ojengbede OA, Meyer CD, Fathalla M, Morhason-Bello IO, Galadanci H, et al. Can the Non-pneumatic Anti-Shock Garment (NASG) reduce adverse maternal outcomes from postpartum hemorrhage? Evidence from Egypt and Nigeria. Reproductive health. 2010;7:24.
6	Miller S, Ojengbede O, Turan JM, Morhason-Bello IO, Martin HB, Nsima D. A comparative study of the non-pneumatic anti-shock garment for the treatment of obstetric hemorrhage in Nigeria. International journal of gynaecology and obstetrics: the official organ of the International Federation of Gynaecology and Obstetrics. 2009;107 (2):121–5.
7	El Ayadi A, Gibbons, L., Bergel, E., Butrick, E., Huong, N.M., Mkumba, G., Kaseba, C., Magwali, T., Merialdi, M., Miller, S. Per-protocol effect of erlier non-pneumatic anti-shock garment application for obstetric hemorrhage. Brief communication. 2014
8	Miller S, Turan JM, Dau K, Fathalla M, Mourad M, Sutherland T, et al. Use of the non-pneumatic anti-shock garment (NASG) to reduce blood loss and time to recovery from shock for women with obstetric haemorrhage in Egypt. Global public health. 2007;2 (2):110–24.
**Descriptive studies, without comparison between NASG and standard treatment for PPH**	
1	El Ayadi A, Raifman S, Jega F, Butrick E, Ojo Y, Geller S, et al. Comorbidities and lack of blood transfusion may negatively affect maternal outcomes of women with obstetric hemorrhage treated with NASG. PloS one. 2013;8 (8):e70446.
2	El Ayadi AM, Butrick E, Geissler J, Miller S. Combined analysis of the non-pneumatic anti-shock garment on mortality from hypovolemic shock secondary to obstetric hemorrhage. BMC pregnancy and childbirth. 2013;13:208.
3	Kausar F, Morris JL, Fathalla M, Ojengbede O, Fabamwo A, Mourad-Youssif M, et al. Nurses in low resource settings save mothers’ lives with non-pneumatic anti-shock garment. MCN The American journal of maternal child nursing. 2012;37 (5):308–16.
4	Lester F, Stenson A, Meyer C, Morris J, Vargas J, Miller S. Impact of the Non-pneumatic Antishock Garment on pelvic blood flow in healthy postpartum women. American journal of obstetrics and gynecology. 2011;204 (5):409 e1-5.
5	Berdichevsky K, Tucker C, Martinez A, Miller S. Acceptance of a new technology for management of obstetric hemorrhage: a qualitative study from rural Mexico. Health care for women international. 2010;31 (5):444–57.
**Serious limitation on risk of bias (only summary available, incomplete data on results of outcomes of interest without explanation)**	
1	Mkumba G, Butrick, E., Amafumba, R., McDonald, K., DeMulder, J., El Ayadi, A., Lippman, S., Gibbons, L., Bergel, E., Merialdi, M., Miller, S. Non-pneumatic anti-shock garment (NASG) decreases maternal deaths in Lusaka, Zambia. International journal of gynaecology and obstetrics: the official organ of the International Federation of Gynaecology and Obstetrics. 2012; Free communication (oral) presentations:S424.

The five observational studies were considered as providing low quality evidence due to the study design and the cRCT was considered as providing moderate quality evidence (this study was downgraded due to imprecision of findings, few number of events occurred. The overall quality assessment is presented in the GRADE table (Table 3). All data pooled were from observational studies and a comparison was made with the result of the cRCT on reporting the effect of NASG use for treating postpartum haemorrhage.

**Table 3:** **GRADE table for guiding evaluation of quality of evidence and strength of the recommendation**

**Quality assessment**							**No of patients**		**Effect**		**Quality**	**Importance**
**No of studies**	**Design**	**Risk of bias**	**Inconsistency**	**Indirectness**	**Imprecision**	**Other considerations**	**NASG**	**Standard care**	**Relative (95% CI)**	**Absolute**		
**Maternal deaths - Non-Randomised clinical trials**												
5	observational studies	no serious risk of bias	no serious inconsistency	no serious indirectness	no serious imprecision	none	46/1274 (3.6%)	72/1056 (6.8%)	RR 0.52 (0.36 to 0.77)	33 fewer per 1000 (from 16 fewer to 44 fewer)	⊕⊕ΟΟ LOW	CRITICAL
								2.3%		11 fewer per 1000 (from 5 fewer to 15 fewer)		
**Maternal deaths - Cluster Randomized Trials**												
1	randomised trial	no serious risk of bias	no serious inconsistency	no serious indirectness	Serious^1^	none	4/405 (1%)	11/475 (2.3%)	RR 0.43 (0.14 to 1.33)	13 fewer per 1000 (from 20 fewer to 8 more)	⊕⊕⊕Ο MODERATE	CRITICAL
								2.3%		13 fewer per 1000 (from 20 fewer to 8 more)		
**Severe Maternal Outcome (Severe Morbidity + Deaths)**												
4	observational studies	no serious risk of bias	no serious inconsistency	no serious indirectness	no serious imprecision	none	17/1167 (1.5%)	44/1055 (4.2%)	RR 0.33 (0.19 to 0.57)	28 fewer per 1000 (from 18 fewer to 34 fewer)	⊕⊕ΟΟ LOW	IMPORTANT
								3.2%		21 fewer per 1000 (from 14 fewer to 26 fewer)		
**Severe Maternal Outcome (Severe Morbidity + Deaths) - Before and After Studies**												
3	observational studies	no serious risk of bias	no serious inconsistency	no serious indirectness	no serious imprecision	none	13/764 (1.7%)	32/590 (5.4%)	RR 0.31 (0.17 to 0.59)	37 fewer per 1000 (from 22 fewer to 45 fewer)	⊕⊕ΟΟ LOW	IMPORTANT
								4.7%		32 fewer per 1000 (from 19 fewer to 39 fewer)		
**Severe Maternal Outcome (Severe Morbidity + Deaths) - Cluster Randomized Trials**												
1	randomised trials	no serious risk of bias	no serious inconsistency	no serious indirectness	Serious^1^	none	4/403 (0.99%)	12/465 (2.6%)	RR 0.38 (0.13 to 1.18)	16 fewer per 1000 (from 22 fewer to 5 more)	⊕⊕⊕Ο MODERATE	IMPORTANT
								2.6%		16 fewer per 1000 (from 23 fewer to 5 more)		
**Blood transfusion (ever)**												
5	observational studies	no serious risk of bias	no serious inconsistency	no serious indirectness	no serious imprecision	none	970/1467 (66.1%)	791/1333 (59.3%)	RR 1.02 (0.92 to 1.12)	12 more per 1000 (from 47 fewer to 71 more)	⊕⊕ΟΟ LOW	CRITICAL
								71.1%		14 more per 1000 (from 57 fewer to 85 more)		
**Blood transfusion (ever) - Before and After Studies**												
4	observational studies	no serious risk of bias	no serious inconsistency	no serious indirectness	no serious imprecision	none	803/1069 (75.1%)	623/898 (69.4%)	RR 0.99 (0.97 to 1.02)	7 fewer per 1000 (from 21 fewer to 14 more)	⊕⊕ΟΟ LOW	IMPORTANT
								75.9%		8 fewer per 1000 (from 23 fewer to 15 more)		
**Blood transfusion (ever) - Cluster Randomized Trials**												
1	randomised trials	no serious risk of bias	no serious inconsistency	no serious indirectness	no serious imprecision	none	167/398 (42%)	168/435 (38.6%)	RR 1.09 (0.92 to 1.28)	35 more per 1000 (from 31 fewer to 108 more)	⊕⊕⊕⊕ HIGH	IMPORTANT
								38.6%		35 more per 1000 (from 31 fewer to 108 more)		

^1^Very few events.

**Figure 2:**
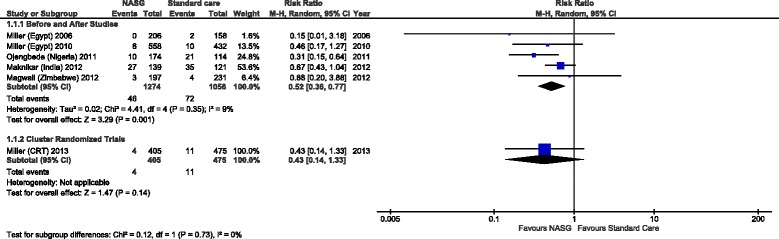
**Pooled analysis comparing the effect of NASG use with standard care to prevent maternal mortality.**

In Figure 2 the pooled analysis for measuring effect of NASG use to prevent maternal mortality is presented. A significant reduction of maternal deaths was seen with NASG use compared to standard treatment, with a pooled risk ratio (RR) and 95% confidence interval of 0.52 (95% CI: 0.36-0.77), five observational studies with NASG group: 1274 women and 46 maternal deaths; and standard care group: 1056 women and 72 maternal deaths. The cRCT showed a non-significant reduction in maternal death in favour of NASG use, RR 0.43 (95% CI: 0.14-1.33); NASG group: 405 women and 4 maternal deaths; and standard care group: 475 women and 11 maternal deaths.

**Figure 3:**
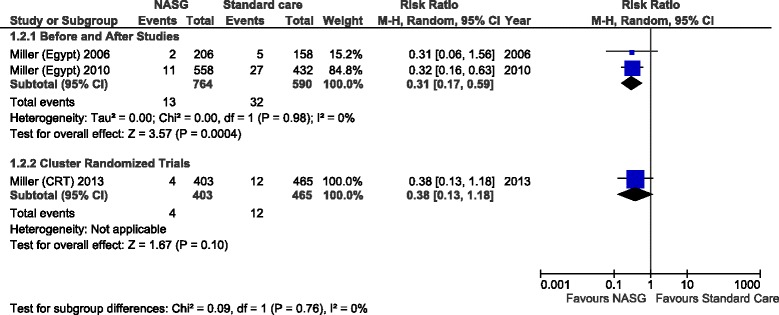
**Pooled analysis comparing the effect of NASG use with standard care to prevent severe maternal outcomes.**

In Figure 3 the outcome of interest was severe maternal complication. The pooled analyses of two observational studies showed a significant reduction on severe maternal outcomes occurrence showing benefit of NASG use, RR 0.31 (95% CI: 0.17 - 0.59), NASG group: 764 women and 13 cases of severe maternal outcomes; standard group: 590 women and 32 cases of severe maternal outcomes. A non-significant trend towards a beneficial effect of NASG use for severe maternal outcomes was also observed in the analysis of c-RCT, RR 0.38 (95% CI: 0.13-1.18); NASG group: 403 women and 4 cases of severe maternal outcomes; standard group: 465 women and 12 cases of severe maternal outcomes.

**Figure 4:**
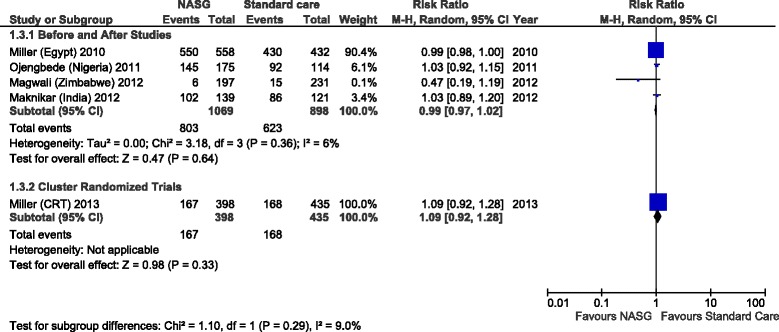
**Pooled analysis comparing the effect of NASG use with standard care to prevent blood products transfusion.**

When NASG use was compared with standard care to prevent blood products transfusion no effect was shown, forest plot for 4 observational studies and for the c-RCT is presented in Figure 4. The pooled analysis of observational studies shows a RR of 0.99 (95% CI: 0.97-1.02); NASG group: 1069 women and 803 women receiving blood transfusion; standard group: 898 women and 623 women receiving blood transfusion. The RR from the c-RCT was 1.09 (95% CI: 0.92-1.28); NASG group: 398 women and 167 women receiving blood transfusion; standard group: 435 women and 168 women receiving blood transfusion.

## Discussion

This systematic review reports on the effects of NASG use as part of PPH management. By systematically reviewing the available scientific evidence, we found that NASG may be able to temporarily mitigate the effects of blood loss after delivery. Among observational studies, NASG was associated with reductions in maternal mortality, compared to standard treatment. There were fewer maternal deaths in NASG group in the randomized controlled trial, but the results were not statistically significant. Despite maternal mortality globally is counted in great figures, at local level; in a health facility, it may represent a rare event. Severe maternal outcomes, such postpartum haemorrhage, blood products use, hypotension were used as proxies for maternal deaths. With maternal death proxies or the presence of any severe maternal complication used as outcome of interest in this systematic review, a similar pattern was observed supporting the trend towards beneficial effects associated with NASG use. However the use of this device did not show an association with reduction of blood transfusion, which suggests that NASG was able to prolong survival until definitive treatment and more resources, such as blood products, become available.

Our findings corroborate the theoretical basis for NASG use: once the systemic blood pressure is maintained in normal ranges for longer periods, progression in the organ dysfunction pathway (i.e. the sequence of blood loss, hypotension, hypovolemic shock, multiple organ failure and death) may be interrupted or delayed. An experimental study suggests a significant increase in internal iliac artery resistive index with NASG application in a small group of postpartum volunteers, which may provide a physiological explanation of how the NASG might reduce postpartum haemorrhage [24]. It should be noted that studies reporting on potential effects of an intervention on maternal mortality are very rare, which underlines the importance of this device and body of knowledge. NASG seems a promising device to be used in developing countries for preventing maternal deaths as it allows additional time of hemodynamic stability for referral and transfer to higher complexity level of facility. An important characteristic of the NASG is the easy use without need of major requirements or training personnel to be included in standard care for PPH. The device is a non-inflatable garment that produces circumferential pressure on the lower extremities and abdomen with no need of tubes, pumps, valves or gauges. The pressure is performed through the elasticity of the neoprene material maintained by the Velcro. The relatively low cost of this device, together with the possibility of reuse makes this device an attractive option for mitigating PPH effects (around USD 50 to 65 per unit) [8]. A cost-effectiveness assessment of NASG use in tertiary hospitals in Egypt and Nigeria found an important improvement in health outcomes at very low costs for treating severe haemorrhagic shock [25]. But it should also be noted that there are some complexities associated with NASG use. These include difficulties to ensure safe re-use (i.e. appropriate cleaning after use and the number of re-uses is possible without loss of compressibility); secure storage after use and availability at site of use of adequate size to fit to anthropometric variation of populations. Another limitation is that this intervention is not definitive: the need for substantive PPH treatment (such as uterotonics) remains.

This systematic review has some strengths and weaknesses that should be acknowledged. It used a comprehensive literature search strategy without limitation of language and databases and research authors were contacted for including unpublished data. Nevertheless, the retrieved evidence is dominated by observational studies, which rendered mostly evidence of low quality due to inherent research design limitations. Additional high quality studies are welcome to assess NASG use, particularly those performed by other research groups in different settings. All studies included in this systematic review were developed by the same research group, five in African countries and one in India. Diversification of scientific experience with NASG would contribute to explore efficacy, safety and applicability in different settings. Knowledge gaps that need to be addressed include the scalability and sustainability of this intervention and the impact that generalized use of NASG may have in strengthening the capacity of health systems to provide definitive PPH treatment. It could be a potential problem if countries prioritize investments in temporizing measures at the expense of more definitive PPH treatments. Other aspects that need to be addressed are related to the expansion of manufacture capacity for NASG and harmonization of clinical pathways to include NASG and with other temporizing measures.

## Conclusion

NASG is a temporizing measure in PPH management potentially able to reduce PPH related deaths and severe morbidities. In settings where delays in PPH management are common, particularly where constraints to offer blood products and definitive treatment exist, use of NASG is an intervention that should be considered as policy option while the conditions for optimal PPH care are created. It is highly recommended that health programs adopting NASG implement a careful monitoring and evaluation strategy to assess the impact of NASG use in PPH management.
